# Zolpidem Activation of Alpha 1-Containing GABA_A_ Receptors Selectively Inhibits High Frequency Action Potential Firing of Cortical Neurons

**DOI:** 10.3389/fphar.2018.01523

**Published:** 2019-01-09

**Authors:** Elena Neumann, Uwe Rudolph, Daniel E. Knutson, Guanguan Li, James M. Cook, Harald Hentschke, Bernd Antkowiak, Berthold Drexler

**Affiliations:** ^1^Experimental Anesthesiology Section, Department of Anesthesiology and Intensive Care, Eberhard Karls Universität Tübingen, Tübingen, Germany; ^2^Department of Comparative Biosciences, College of Veterinary Medicine, University of Illinois at Urbana–Champaign, Urbana, IL, United States; ^3^Department of Chemistry and Biochemistry, University of Wisconsin–Milwaukee, Milwaukee, WI, United States; ^4^Werner Reichardt Center for Integrative Neuroscience, Eberhard Karls Universität Tübingen, Tübingen, Germany

**Keywords:** zolpidem, benzodiazepines, GABA_A_ receptor subtypes, neocortex, electrophysiology, GABA_A_ receptor subtype specific drugs, neocortical activity

## Abstract

**Introduction:** High frequency neuronal activity in the cerebral cortex can be induced by noxious stimulation during surgery, brain injury or poisoning. In this scenario, it is essential to block cortical hyperactivity to protect the brain against damage, e.g., by using drugs that act as positive allosteric modulators at GABA_A_ receptors. Yet, cortical neurons express multiple, functionally distinct GABA_A_ receptor subtypes. Currently there is a lack of knowledge which GABA_A_ receptor subtypes would be a good pharmacological target to reduce extensive cortical activity.

**Methods:** Spontaneous action potential activity was monitored by performing extracellular recordings from organotypic neocortical slice cultures of wild type and GABA_A_R-α1(H101R) mutant mice. Phases of high neuronal activity were characterized using peri-event time histograms. Drug effects on within-up state firing rates were quantified via Hedges’ g.

**Results:** We quantified the effects of zolpidem, a positive modulator of GABA_A_ receptors harboring α1-subunits, and the experimental benzodiazepine SH-053-2′F-S-CH3, which preferably acts at α2/3/5- but spares α1-subunits. Both agents decreased spontaneous action potential activity but altered the firing patterns in different ways. Zolpidem reduced action potential firing during highly active network states. This action was abolished by flumazenil, suggesting that it was mediated by benzodiazepine-sensitive GABA_A_ receptors. SH-053-2′F-S-CH3 also attenuated neuronal activity, but unlike zolpidem, failed to reduce high frequency firing. To confirm that zolpidem actions were indeed mediated via α1-dependent actions, it was evaluated in slices from wild type and α(H101R) knock-in mice. Inhibition of high frequency action potential firing was observed in slices from wild type but not mutant mice.

**Conclusion:** Our results suggest that during episodes of scarce and high neuronal activity action potential firing of cortical neurons is controlled by different GABA_A_ receptor subtypes. Exaggerated firing of cortical neurons is reduced by positive modulation of α1-, but not α2/3/5-subunit containing GABA_A_ receptors.

## Introduction

High frequency electroencephalographic activity is generated during sensory processing and cognition. In human subjects and in rodents sedation and anesthetic-induced unconsciousness correlates with a decrease in high frequency oscillations in the cerebral cortex and the hippocampus (Sleigh et al., 2001; Ma et al., 2002; Hudetz et al., 2011). In patients suffering from traumatic brain injury, exaggerated neuronal activity may lead to post-traumatic epilepsy (Cantu et al., 2015). Epileptic seizures *per se* are characterized by hypersynchronous high neuronal activity. Excessive neuronal activity causes extensive energy consumption and a breakdown of ion gradients across neuronal membranes. Furthermore, calcium ions enter the cytoplasm through different routes, including extra-synaptic NMDA receptors and internal calcium stores (Bano et al., 2005). These events potentially trigger apoptotic pathways, possibly resulting in long-lasting cognitive impairment (Parsons and Raymond, 2014). In these scenarios it seems desirable to limit cortical hyperactivity to prevent further damage. Most drugs currently used to depress cortical activity such as anesthetics or benzodiazepines act on the molecular level by enhancing the function of GABA_A_ receptors. In cortical networks multiple GABA_A_ receptor subtypes are abundant (Rudolph et al., 2001; Sieghart and Sperk, 2002; Whiting, 2003; Möhler, 2006). They differ in their subunit composition, cellular localization, and physiological function. However, it remains to be elucidated to what extent distinct receptor subtypes are involved in the control of high frequency neuronal activity.

In the cerebral cortex about 80% of neurons are glutamate-releasing PCs, which innervate different classes of GABAergic interneurons. About half of all GABA_A_ receptors in the cerebral cortex harbor α1-subunits. They reside at high densities at the somata of PCs. Since action potentials are generated at the somata and axon hillock, the presence of α1-GABA_A_ receptors at somatic sites makes it likely that this receptor subtype plays a major role in the control of action potential generation. We, therefore, hypothesized that high frequency action potential firing of cortical neurons can be attenuated by drugs which enhance the function of α1-subunits containing GABA_A_ receptors. Consequently, we examined the effects of zolpidem, a positive modulator of α1-containing GABA_A_ receptors (Sanna et al., 2002; Baur and Sigel, 2007). Studies on expressed receptors suggest that zolpidem’s selectivity for α1-receptors is limited to low concentrations. To clearly distinguish the drug’s action at α1-receptors from effects via other targets, we studied zolpidem in slices prepared from both wild type and α1(H101R) knock-in mice (Rudolph et al., 1999). In these mutants, the physiological function of GABA_A_ receptors harboring α1-subunits is not substantially compromised, but receptors are largely resistant to modulation by positive allosteric modulators of the benzodiazepine binding site, including zolpidem. To confirm the specificity of results obtained by α1-containing GABA_A_ receptors preferring zolpidem in wild type and α1(H101R) cultured slices, we also tested the α2/3/5-preferring experimental benzodiazepine SH-053-2′F-S-CH3 (Fischer et al., 2010; Savic et al., 2010; Richetto et al., 2015). The major result is that positive modulation of α1-, but not α2/3/5-containing GABA_A_ receptors effectively reduces action potential firing during phases of exaggerated neuronal activity.

## Materials and Methods

### Animals

For this study, we used 12 mice of both sexes, homozygous for an histidine to arginine point mutation at position 101 of the GABA_A_ receptor α1-subunit (H101R) and 50 homozygous wild type controls of the same genetic background (C57BL/6J) (Rudolph et al., 1999). All procedures were approved by the Animal Care Committee (Eberhard-Karls-University, Tuebingen, Germany) and were in accordance with the institutional and federal guidelines of the German Animal Welfare Act (TierSchG). We put in a great deal of effort to reduce the number and suffering of animals.

### Organotypic Slice Cultures and Electrophysiology

**FIGURE 1:**
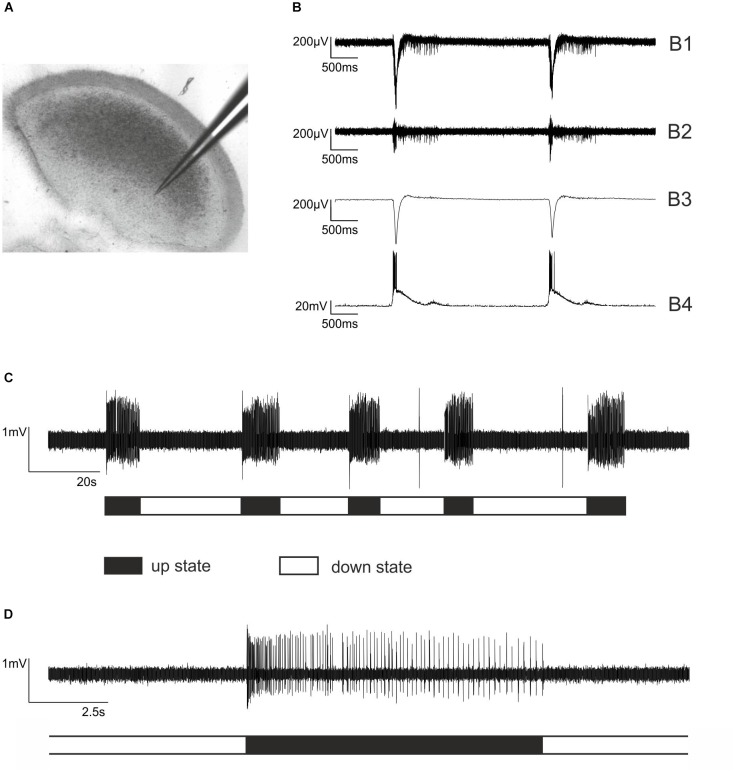
Extracellular recordings from cortical slice cultures and firing patterns of cultured cortical neurons. **(A)** An electrode made of borosilicate glass placed in an organotypic cortical slice culture. Note that the cortical architecture is largely preserved. **(B)** Simultaneous extra- and intracellular recording from a neocortical cultured slice to illustrate the functional properties of the network. **(B1)** Raw trace of extracellular recording. **(B2)** The signal was band pass filtered 250 – 5000 Hz to allow for action potential detection. **(B3)** Local field potential, sometimes referred to as “micro-EEG,” which is obtained from **B1** by low pass filtering 1 – 40 Hz. **(B4)** Simultaneous intracellular current clamp recording from a cortical pyramidal neuron within the network. The liquid junction potential of –17 mV was subtracted. **(C)** The typical firing pattern of cultured cortical neurons is characterized by alternating phases of high neuronal activity, so-called bursts or up states and phases of very low activity, called silent period or down state. Here, five bursts of action potential firing (up states) are displayed, halted by silent periods (down states). **(D)** One single up state at a higher temporal resolution. Each vertical line represents a single action potential. Note that the phase of neuronal activity at the beginning of the up state is rather high, followed by moderate activity that is slowly declining.

We prepared neocortical slice cultures from 2 to 5-day old mice as previously described (Gähwiler, 1981). For the preparation of the somatosensory cortex, mice were deeply anesthetized using isoflurane and decapitated. Cortical hemispheres were aseptically removed and after removal of the meninges, coronal slices were cut. These slices were fixed on glass coverslips using a plasma clot. Slices were cultured with nutrition medium in a roller drum at 37°C. For the current study we performed 305 extracellular network recordings of single- and multi-unit cortical activity in 173 slice cultures from wild type mice and 140 recordings in 81 cultures from α1(H101R) knock-in mice at 15 to 35 days *in vitro*. Recordings were performed with one or two extracellular electrodes in a recording chamber mounted on an inverted microscope (Figure 1A). Further, we perfused slices with ACSF, consisting of (in mM) NaCl 120, KCl 3.3, NaH_2_PO_4_ 1.13, NaHCO_3_ 26, CaCl_2_ 1.8 and glucose 11, bubbled with 95% oxygen and 5% carbon dioxide. We advanced ACSF-filled glass electrodes with a resistance of about 3 to 5 MΩ into the tissue until extracellular spikes exceeding 100 μV in amplitude were visible. We conducted all experiments at 34°C and acquired data on a personal computer, using a digidata 1200 AD/DA interface and Axoscope 9 software (Axon Instruments, Foster City, CA, United States). For Figure 1B4 whole-cell current clamp recording was performed from a visually identified pyramidal neuron. A borosilicate electrode was filled with a solution containing (in mM) K-gluconate 135, HEPES 10, EGTA 10, CaCl_2_ 0.5, MgCl_2_ 2.0, Na_2_ATP 3.0, NaGTP 0.3, Na_2_phosphocreatine 10.0 (pH 7.3), and had a resistance of ∼3.5 MΩ. The signal was acquired with a MultiClamp 700B amplifier, lowpass filtered at 6000 Hz, and digitized at 20 kHz via a Digidata 1440 interface and pClamp 10 software (Molecular Devices, San Jose, CA, United States).

### Preparation and Application of Test Solutions

We prepared a stock solution of zolpidem and flumazenil (both from Sigma, Taufkirchen, Germany) by diluting them in ethanol, while preparing a stock solution of SH-053-2′F-S-CH3, by diluting it in dimethyl sulfoxide. All drug-containing solutions were frozen, freshly dissolved in ACSF to the desired concentrations and filled into gastight glass syringes. The ethanol and dimethyl sulfoxide dilutions used for the current study had no effect on the firing patterns of cultured neocortical neurons (Drexler et al., 2013).

A drug containing ACSF was applied via bath perfusion, using syringe pumps (ZAK, Marktheidenfeld, Germany) connected to the experimental chamber via Teflon tubing (Lee, Frankfurt, Germany). The flow rate was approximately 1 ml min^-1^. After switching from ACSF to drug-containing solutions, we replaced the medium in the experimental chamber by at least 95% within 2 min. To ensure steady state conditions, we performed recordings during drug treatment in 10-min intervals after changing the perfusate. This time interval proved to be sufficient for steady state conditions (Antkowiak, 1999), as diffusion times in slice cultures are considerably shorter compared to acute slice preparations (Gredell et al., 2004; Benkwitz et al., 2007). Slices were exposed to a maximum of two concentrations of the drug tested.

### Data and Statistical Analysis

We estimated group sizes based on earlier studies and analyzed recorded signals offline, using self-written programs in MATLAB (The MathWorks Inc., Natick, MA, United States). The typical firing pattern of neocortical slice cultures is characterized by bursts of spontaneous action potential firing (up state) separated by periods of neuronal silence (down state, see Figure 1C). An up state was defined as a group of action potentials with an initially high firing rate of 10 Hz, preceded by a down state. For further analysis, action potential activity in single cultures was binned and firing rates were averaged over all up states sampled during the standard recording interval of 180 s. We calculated grand averages, using data from different experiments to control unwanted sources of variation. Subsequently, we calculated PETHs by using time bins of 5 ms where *t* = 0 marks the beginning of the burst.

For statistical quantification of bin-wise firing rates in PETHs, we report an effect statistic, Hedges’ g, which is a standardized difference between two means, including 95% CIs (Nakagawa and Cuthill, 2007; Hentschke and Stuttgen, 2011). The higher the absolute value of *d*, the stronger the effect, and in cases in which the 95% CI does not encompass zero the effect can be considered significant (*p* < 0.05) within the framework of hypothesis testing (Nakagawa and Cuthill, 2007). All results except for *d* are presented as mean ± SEM or as median and iqr, as appropriate. For comparison of drug versus control condition of all other parameters, we used the Mann–Whitney *U*-test followed by Bonferroni-Holm correction.

## Results

### Cortical Firing Patterns Under Control Conditions and in the Presence of Bicuculline and Isoguvacine

**FIGURE 2:**
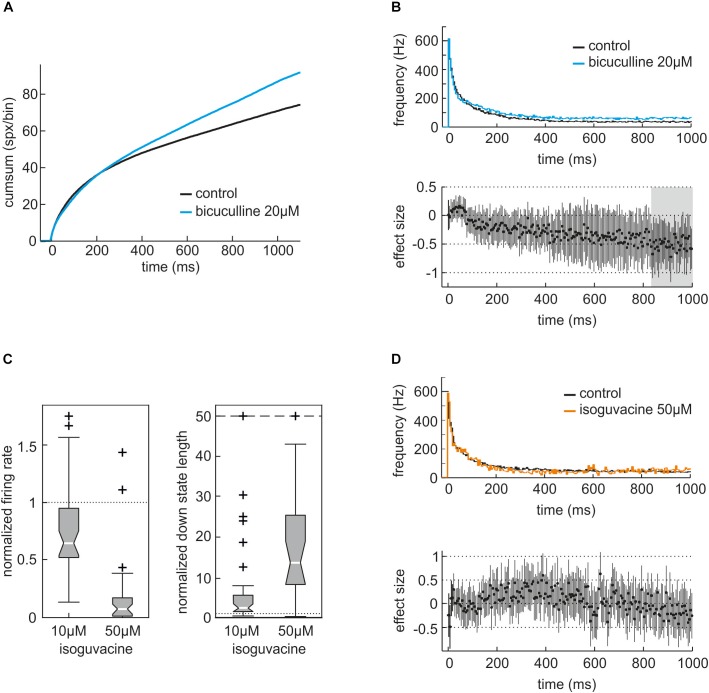
Actions of the GABA_A_ receptor antagonist bicuculline and the GABA_A_ receptor agonist isoguvacine on neuronal activity of neocortical slice cultures. **(A)** Cumulative sum of action potentials during the first 1000 ms of the averaged up state in the absence (control, black) and presence of 20 μM bicuculline (blue, *n* = 32). **(B)** Peri-event time histogram (PETH) of averaged neuronal up states in the absence (black) and presence of bicuculline (blue). For the analysis of actions of GABAergic modulators on neuronal activity we collected all up states, averaged and divided these into time bins of 5 ms. Then, we calculated the instantaneous frequency of action potential firing for each 5-ms time bin and plotted them against the time. In the presence of 20 μM bicuculline, activity is almost constantly higher compared to control condition. Lower part of the figure: effect size. An effect size of one would indicate that the neuronal activity is one standard deviation apart. A significant difference between the two conditions can be assumed if the 95% confidence interval (CI) does not cross the zero line. Here, the effect of bicuculline is slightly building up and most prominent after 800 ms from the beginning of the up state, marked by gray shadowing. **(C)** Actions of 10 and 50 μM isoguvacine on the normalized firing rate and the normalized length of down states given as boxplot. Isoguvacine depressed the averaged action potential firing rate to 0.64 (median, iqr = 0.42, *n* = 40, *p* = 0.00012, Mann–Whitney *U*-test compared to control condition) at 10 μM and to 0.07 (median, iqr = 0.16, *n* = 25, *p* < 0.0001) at 50 μM. Meanwhile, the averaged length of neuronal down states was prolonged by 10 μM isoguvacine to 2.59 (median, iqr = 4.03, *n* = 40, *p* < 0.0001) and to 13.66 (median, iqr = 17.1, *n* = 25, *p* = 0.000012) at a concentration of 50 μM. **(D)** Analysis of the actions of 50 μM isoguvacine within neuronal up states given as PETH plus corresponding effect size. The PETH of control (black) and 50 μM isoguvacine (orange) are virtually superimposable while the corresponding effect size swings round the zero line, indicating that isoguvacine does not primarily affect neuronal up states. Taken together this illustrates that isoguvacine is depressing neuronal activity in neocortical cultures from mice by prolonging neuronal down states while activity within up states is almost unaffected.

Typical neocortical activity patterns under drug-free conditions are displayed in Figures 1B–D. The dynamics of and the relation between population and single-neuron activity in the isolated cortical micro-network are exemplarily illustrated in Figure 1B, showing a combined extra- and intracellular recording from a cultured cortical slice. Single PC action potential firing (Figure 1B4) occurs synchronously with the appearance of up states of the network. In Figure 1C five up states separated by neuronal silence (down states) are visible. Figure 1D shows a single up state at higher temporal resolution. At the beginning of an up state, discharge rates are high, but then rapidly decline. To uncover the actions of interference within the GABAergic system we performed experiments with the GABA_A_ receptor antagonist bicuculline and the GABA_A_ receptor agonist isoguvacine. Bicuculline at a concentration of 20 μM led to an accumulative increase of neuronal activity in neocortical cultures, slowly but continuously raising the activity a few hundred milliseconds after the beginning of neuronal up states (Figures 2A,B). By contrast, the GABA_A_ receptor agonist isoguvacine depressed neuronal activity mainly by substantially prolonging the phases of neuronal silence (Figures 2C,D). Taken together these data illustrate that interventions within the cortical GABAergic system lead to differential and specific effects on neuronal firing patterns.

**FIGURE 3:**
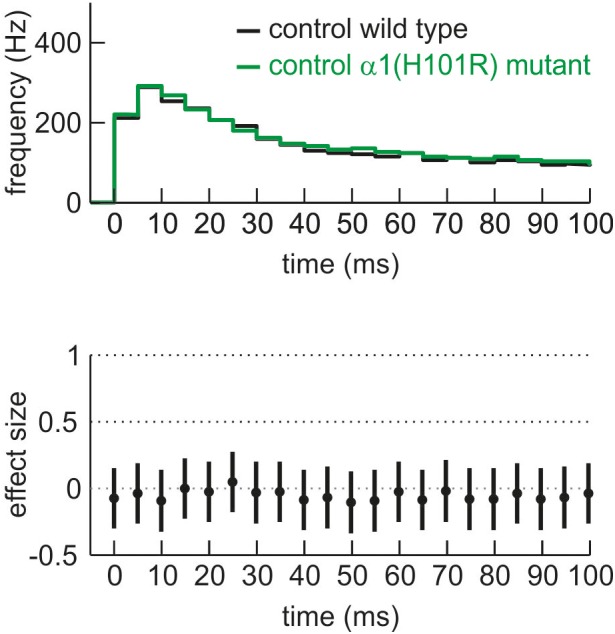
Comparison of neuronal activity of cultured cortical neurons derived from wild type (black) and α1(H101R) mutant mice (green) given as PETH and corresponding effect size for the first 100 ms of neuronal up state. There is no difference in neuronal activity under control conditions between wild type and α1(H101R) mutant cortical circuits.

For the current study we aimed at detecting zolpidem’s α1-mediated actions by comparing its effects in slices derived from wild type and α1(H101R) knock-in mice in which zolpidem does not bind to α1-containing GABA_A_ receptors (Rudolph et al., 1999). However, this approach raised the question whether the mutation *per se* alters the firing properties of cortical neurons under drug-free conditions. Figure 3 displays the PETH of firing rates in slices derived from wild type and α1(H101R) mutant mice during an interval of 100 ms following the onset of up states. During this time window, neurons discharge at their maximal frequencies. For statistical comparison we calculated effect sizes (Hedges’ g) and 95% CIs for corresponding bins. Here, the GABA_A_ receptor α1(H101R) point-mutation did not alter the discharge rate under drug free conditions. For additional confirmation that the mutation was silent, we calculated the frequency of action potentials, which we sampled during the total recording period of 180 s. This measure depends on the firing rates within up states and on the duration of up and down states. The mean action potential firing rate under drug free conditions was 14.6 ± 0.9 Hz (*n* = 136) for wild type slices and 14.7 ± 0.9 Hz (*n* = 81, *p* > 0.5) for α1(H101R) mutant mice slices, providing further evidence that the mutation did not alter the discharge properties of neocortical neurons.

### Zolpidem Depresses Peak Firing Rates in Slices From Wild Type, but Not From α1(H101R) Mutant Mice

**FIGURE 4:**
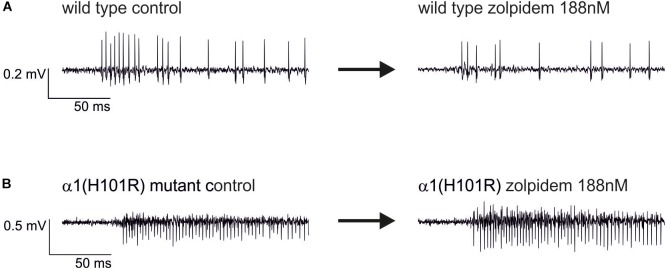
Original extracellular recordings from neocortical cultures of wild type **(A)** and α1(H101R) mutant mice **(B)** under control conditions (left traces) and in the additional presence of 188 nM zolpidem (right traces). The depression of spontaneous neuronal activity by zolpidem right after the onset of the up state is more pronounced in the wild type.

**FIGURE 5:**
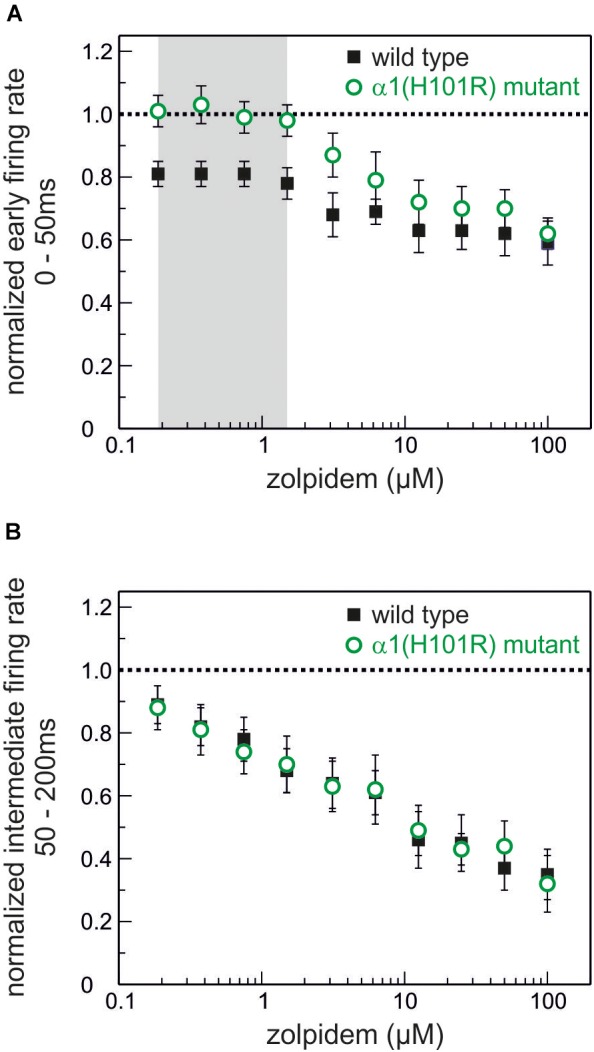
Actions of the α1-GABA_A_ receptor-preferring drug zolpidem on neuronal activity. **(A)** Effects of zolpidem (concentration range 188 nM – 100 μM) on the normalized action potential rate during phases of high neuronal activity (0 – 50 ms after the beginning of an up state) in wild type (black) and GABA_A_ α1(H101R) (green) cultured cortical neurons. The network-depressant action of zolpidem at low concentrations (up to 1.5 μM, marked in gray) is absent in neurons from GABA_A_ α1(H101R) mice. Starting at a concentration of 3.125 μM inhibition of action potential firing during phases of high neuronal activity can be observed in GABA_A_ α1(H101R) neurons and at higher concentrations runs parallel to the effects in wild type neurons (mean ± SEM, *n* = 22 – 46 for wild type and 8 – 38 for α1(H101R) mutant). **(B)** Effects of zolpidem on intermediate neuronal activity (during the time window of 50 – 200 ms after the beginning of an up state). Zolpidem depresses neuronal activity in a concentration-dependent manner in both wild type (black) and GABA_A_ α1(H101R) (green) cortical neurons (mean ± SEM, *n* = 16 – 41 for wild type and 12 – 34 for α1(H101R) mutant). Unlike in **A** (high neuronal activity 0 – 50 ms after up state initiation) there is no difference between the action of zolpidem in wild type and GABA_A_ α1(H101R) neurons. Data is displayed as mean ± SEM.

Original traces of the actions of zolpidem (188 nM) in wild type and α1(H101R) mutant cultured slices are given in Figure 4. Figure 5 summarizes the concentration-dependent effects of zolpidem on the mean discharge rate in the time intervals 0 – 50 ms (Figure 5A) and 50 – 200 ms (Figure 5B), following up state onset. At concentrations of 188 nM – 1.5 μM zolpidem reduced mean discharge rates only in wild type, but not in α1(H101R) mutant slices (marked by gray shading). However, at higher concentrations inhibition was also evident in slices from α1(H101R) mutant mice, suggesting that the drug increasingly lost its α1-selectivity. Zolpidem also reduced firing rates during a time window of 50 – 200 ms following up state onset. Interestingly, during this phase of neuronal activity depression of action potential firing was almost identical in slices from wild type and α1(H101R) mutant mice, indicating that α1-containing GABA_A_ receptors are of minor or no importance in this phase of neuronal activity.

**FIGURE 6:**
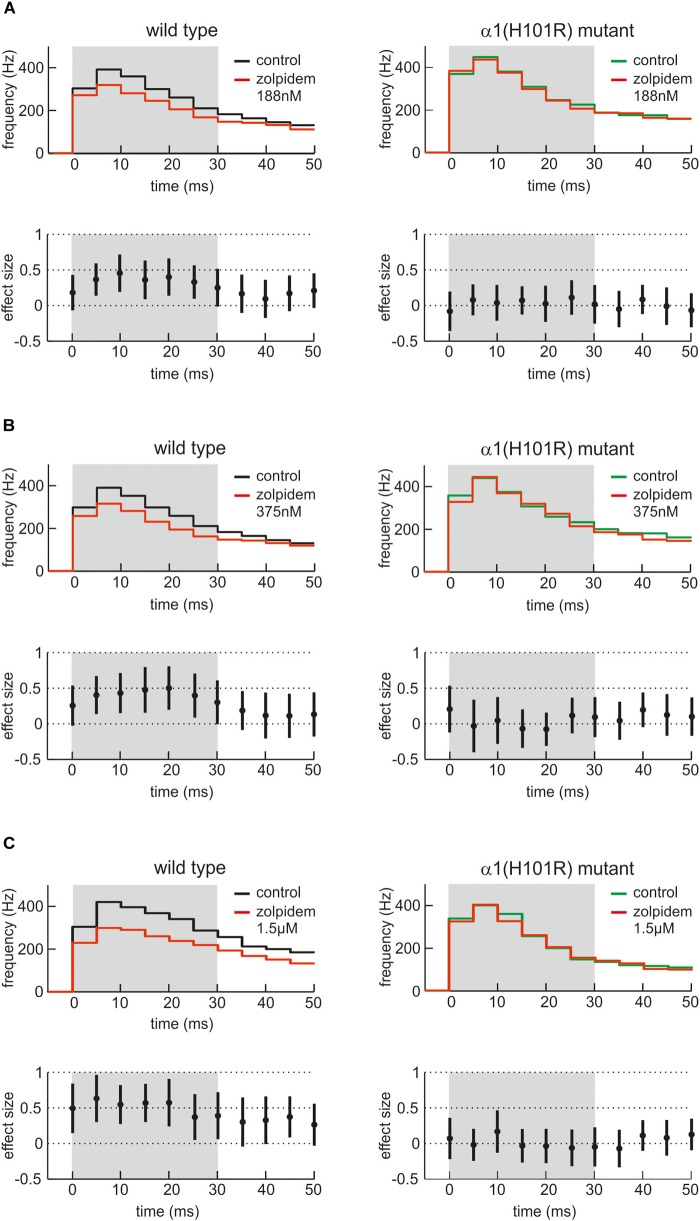
Actions of zolpidem on phases of high neuronal activity. **(A–C)** The effects of three low concentrations of zolpidem (**A**: 188 nM; **B**: 375 nM; **C**: 1.5 μM, *n* = 29 – 41, presence of zolpidem indicated in red) are given as PETH with corresponding effect sizes for wild type cultured cortical neurons (left side, black) and neurons from α1(H101R) mutant mice (right side, green). In each case, the upper half of the figure displays the instantaneous action potential firing rate under control condition and in the presence of zolpidem (red). The lower half of the figure shows the corresponding effect size, with a 95% CI not crossing the zero line being equivalent to a statistically significant effect of zolpidem. To reveal the action of zolpidem, PETH and effect size is shown for the first 50 ms after the beginning of averaged neocortical up states. Note that zolpidem induces a depression of neuronal activity between 5 and 30 ms after the beginning of the up state, i.e., when neuronal activity is particularly high. The effect of zolpidem was absent in neurons derived from α1(H101R) mutant mice (right side of the figure), indicating that the action of zolpidem is indeed mediated via α1-containing GABA_A_ receptors.

Figure 6 presents the effects of three different concentrations of zolpidem in slices from wild type and α1(H101R) mutant mice. In all PETH firing rates peaked at the second bin, i.e., 5 – 10 ms after onset of up states. Zolpidem decreased firing rates in slices from wild type, but not in α1(H101R) mice. Statistically significant differences of discharge rates in the presence and absence of zolpidem in wild type slices are indicated by a gray background. It should be noted that effects are maximal while neurons discharge at their highest rates.

**FIGURE 7:**
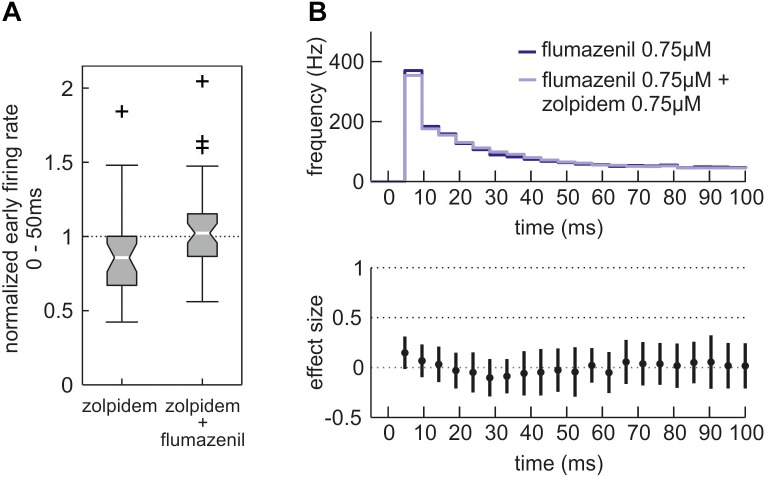
Effects of zolpidem in the additional presence of the benzodiazepine antagonist flumazenil. **(A)** Action of zolpidem (0.75 μM) in the absence and presence of flumazenil (0.75 μM) given as boxplot. While depression of high neuronal activity (0 – 50 ms after initiation of up state) by zolpidem is evident (left side, *n* = 30, median = 0.86, iqr = 0.33 of normalized activity) it is absent in the additional presence of flumazenil (right side, *n* = 47, median = 1.02, iqr = 0.29 of normalized activity, *p* = 0.0147, Mann–Whitney *U*-test). **(B)** Action of zolpidem (0.75 μM) in the presence of flumazenil (0.75 μM) given as PETH, with the dark blue curve showing the neuronal activity in the sole presence of flumazenil and the light blue curve showing a combined application of flumazenil and zolpidem. Within the first 100 ms after the beginning of neuronal up states the depressant action of zolpidem is absent in the additional presence of flumazenil.

To confirm that the effects of zolpidem were mediated via the benzodiazepine binding site of the GABA_A_ receptor, we applied the specific antagonist flumazenil. Figure 7 demonstrates that flumazenil abolished zolpidem effects during phases of high neuronal activity.

### Effects of the α2/3/5-Preferring Benzodiazepine SH-053-2′F-S-CH3

The results presented in Figures 5, 6 provide evidence for an important role of α1-containing GABA_A_ receptors in controlling highly active network states. In a previous study (Drexler et al., 2013) we showed that positive modulation of α5-containing GABA_A_ receptors affected the discharge patterns without altering high frequency action potential firing, suggesting that not all GABA_A_ receptor subtypes may be suitable pharmacological targets for blocking cortical hyperactivity. High affinity binding sites for benzodiazepines are present on GABA_A_ receptors incorporating α1-, α2-, α3-, or α5-subunits. Thus, we wondered how the firing patterns of cortical neurons are affected by modulation of α2/3/5 receptors. It was previously demonstrated that the experimental benzodiazepine SH-053-2′F-S-CH3 preferably acts via α2-, α3-, and α5-containing GABA_A_ receptors (Fischer et al., 2010; Savic et al., 2010), thus having a pharmacological GABA_A_ receptor subtype target profile exactly opposite to that of the α1-preferring zolpidem.

**FIGURE 8:**
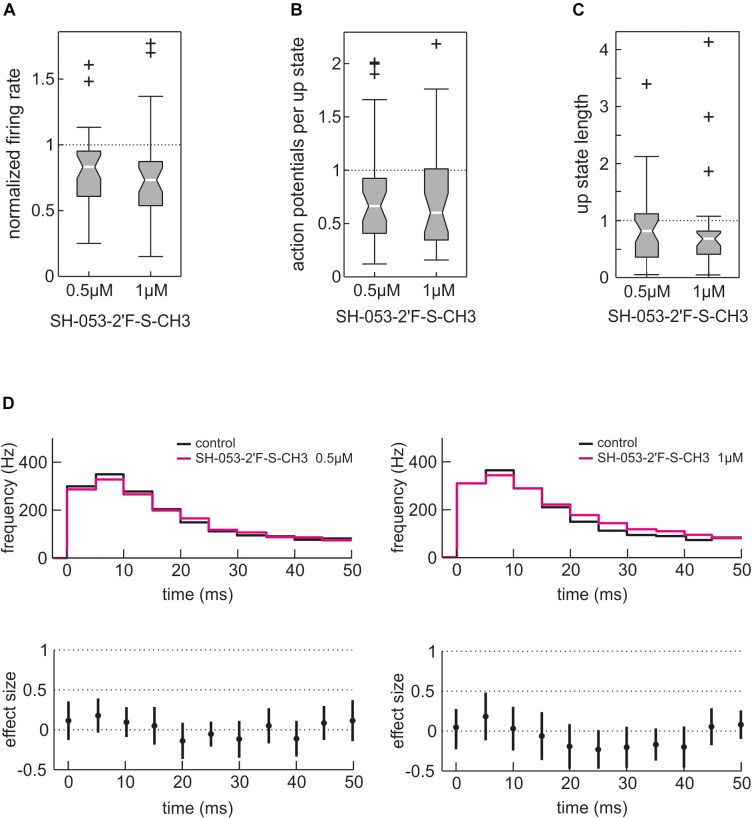
Effect of the α2/3/5 GABA_A_ receptor-preferring experimental benzodiazepine SH-053-2′F-S-CH3 on cortical network activity. Unlike the α1-subtype GABA_A_ receptor-preferring zolpidem, SH-053-2′F-S-CH3 predominantly acts via α2-, α3-, and α5-containing GABA_A_ receptors. **(A)** Action of SH-053-2′F-S-CH3 (0.5 and 1.0 μM) on overall neuronal activity given as boxplot. As expected from a benzodiazepine, SH-053-2′F-S-CH3 depressed cortical activity to 0.83 (median, iqr = 0.34, *n* = 37, *p* = 0.00025, Mann–Whitney *U*-test compared to control condition) at 0.5 μM and to 0.73 of control activity (median, iqr = 0.34, *n* = 33, *p* = 0.0005, Mann–Whitney *U*-test compared to control condition) at 1.0 μM. **(B)** Effect of SH-053-2′F-S-CH3 on the number of action potentials per up state. SH-053-2′F-S-CH3 reduced the number of action potentials per up state to 0.66 of control condition at 0.5 μM (median, iqr = 0.52, *n* = 37, *p* = 0.0074, Mann–Whitney *U*-test) and to 0.60 at 1.0 μM (median, iqr = 0.67, *n* = 33, *p* = 0.012, Mann–Whitney *U*-test). **(C)** Action of SH-053-2′F-S-CH3 on up state length. SH-053-2′F-S-CH3 reduced the median length of neuronal up states to 0.82 of control at 0.5 μM (median, iqr = 0.76, *n* = 37, *p* = 0.0261, Mann–Whitney *U*-test) and to 0.68 (median, iqr = 0.40, *n* = 33, *p* = 0.0013, Mann–Whitney *U*-test compared to control condition) at 1.0 μM. **(D)** The effects of 0.5 μM (*n* = 37) and 1.0 μM (*n* = 33) SH-053-2′F-S-CH3 on the first 50 ms of cortical up states are displayed as PETH, action of SH-053-2′F-S-CH3 in magenta versus control condition in black. Unlike zolpidem, SH-053-2′F-S-CH3 did not induce inhibition during phases of very high cortical activity (0 – 50 ms after the onset of cortical up states). At the higher concentration of 1.0 μM, SH-053-2′F-S-CH3 even tended to induce a slightly higher neuronal activity between 20 and 40 ms after the beginning of the up state (see right lower part of the figure).

As expected from a benzodiazepine, SH-053-2′F-S-CH3 depressed neuronal activity in cortical slice cultures (Figures 8A–C) in a concentration-dependent manner. This network depressing effect was evident for the overall event rate (Figure 8A) and can be explained by SH-053-2′F-S-CH3 reducing the number of action potentials per up state (Figure 8B) and by shortening the up state length (Figure 8C). However, in contrast to zolpidem, SH-053-2′F-S-CH3 (0.5 and 1.0 μM) did not reduce action potential firing during highly active network states observed 0 – 100 ms after up state onset (Figure 8D). Overall, these findings suggest that among benzodiazepine-sensitive GABA_A_ receptors mainly those containing α1-subunits are capable of limiting action potential firing during states of exaggerated neuronal activity.

## Discussion

The neocortex is an important target for benzodiazepines and general anesthetic agents (Heinke et al., 2004; Hentschke et al., 2005). Furthermore, sedation with benzodiazepines predominantly involves cortical α1-containing GABA_A_ receptors located on glutamatergic PCs (Zeller et al., 2008). These receptors also participate in seizure susceptibility (Kralic et al., 2002) and, in turn, mediate anticonvulsive drug actions (Rudolph et al., 1999; Vlainic and Pericic, 2010). To elucidate the role of α1-containing GABA_A_ receptors in shaping cortical activity patterns we opted for an *ex vivo* approach since it prevents active metabolites of the drugs under investigation from compromising the results. In OTCs derived from the rodent neocortex, patterns of neuronal activity are similar to those in the intact cerebral cortex, given that subcortical inputs are absent (Corner, 2008) and the development is similar to the corresponding *in vivo* situation (De Simoni et al., 2003; Di Cristo et al., 2004). Similarity in development particularly includes the hyperpolarizing nature of GABA at the time OTC are used for recordings (Owens et al., 1996; Grasshoff et al., 2008), thereby representing an adult-like status of the neuronal network. The inhibitory nature of GABAergic drugs like intravenous anesthetics and benzodiazepines in cortical OTC has been shown in a number of previous studies, e.g., (Drexler et al., 2009, 2010; Razik et al., 2013; Balk et al., 2017). We, therefore, explored the actions of the GABA_A_ receptor α1-preferring agent zolpidem in cultured slices from wild type and α1(H101) knock-in mice. It had previously been shown that the α1(H101R) point mutation renders this specific GABA_A_ receptor subtype insensitive to benzodiazepines and zolpidem (Rudolph et al., 1999; Crestani et al., 2000).

The key finding from this study is that zolpidem inhibits neuronal activity, especially during phases of high network activity, an effect which is neither observed in neurons from α1(H101R) mutant mice nor is it produced by the α2/3/5-preferring benzodiazepine SH-053-2′F-S-CH3. This observation suggests that the activation of α1-receptors by GABA is largely restricted to episodes of high frequency action potential firing.

### Modulation of Neuronal Network Activity by Interventions in the GABAergic System

The firing patterns displayed in Figure 1 are strongly shaped by GABA_A_ receptor activity. This is indicated by the observation that the specific GABA_A_ receptor antagonist bicuculline evoked rhythmic high frequency firing during bursts (Antkowiak and Hentschke, 1997) and significantly increased average firing rates of cultured cortical neurons about twofold (Grasshoff et al., 2007). Furthermore, bicuculline reduced the modulatory action of GABA-reuptake blockers (Razik et al., 2013) and drugs acting as positive allosteric modulators at GABA_A_ receptors predominantly during bursts (Antkowiak, 1999). In the present study, we re-evaluated the effects of bicuculline (20 μM) in cultured slices in order to confirm these previous reports. Consistent with previously published data, bicuculline enhanced action potential firing rates during neuronal up states (Figures 2A,B).

In addition, we investigated the effects of the GABA_A_ receptor agonist isoguvacine. As expected, this agent depressed overall neuronal activity. However, this effect was mainly due to prolonged phases of neuronal down states, whereas up states remained almost unchanged (Figures 2C,D). These observations are in accordance with the effects of muscimol, another specific GABA_A_-receptor agonist (Razik et al., 2013). In cultured neocortical slices, muscimol prolonged neuronal down states, leaving up state activity almost unaffected. Taken together, the effects of bicuculline, isoguvacine and muscimol provide evidence that action potential firing during neuronal up states on the one hand, and during down states on the other hand is under the control of different types of GABA_A_ receptor-mediated inhibition. Phasic inhibition mediated by synaptic GABA_A_ receptors seems to dominate during neuronal up states, whereas tonic activation of putative extrasynaptic GABA_A_ receptors as caused by isoguvacine and muscimol is most effective during cortical down states (Razik et al., 2013).

### Zolpidem’s α1-Mediated Action Time-Locked to Highly Active Network States

**FIGURE 9:**
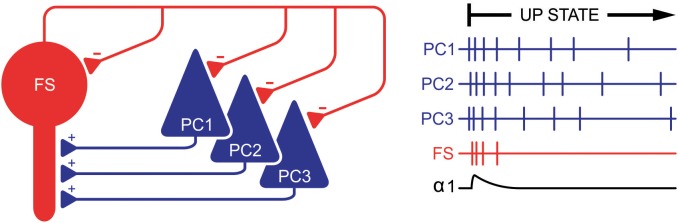
Suggested mechanism that time-locks activation of α1-containing GABA_A_ receptors to highly active network states. Right after the beginning of an up state, pyramidal cells (PCs) generate action potentials (vertical bars on the right) at a high frequency, thereby exciting fast-spiking basket cells (FS). This excitation is only strong and synchronous within the first approximate 50 ms of up states, although sufficient to induce action potential firing of FS cells. These FS cells in turn inhibit PCs via α1-containing GABA_A_ receptors. However, this inhibition is transient since FS cells undergo self-inhibition via autapses, thereby limiting activation of α1-containing GABA_A_ receptors and α1-mediated actions of zolpidem to a narrow time window (lowest trace on the right). Later, excitation of FS cells by PC is weaker and asynchronous, which is insufficient to induce action potential firing in FS cells (and subsequent activation of α1-containing GABA_A_ receptors). During this phase of neuronal activity GABAergic inhibition is provided by non-α1-containing GABA_A_ receptors.

Can the observation that zolpidem’s α1-mediated action is time-locked to highly active network states be explained by current knowledge? Basically, GABA_A_ receptor activation largely depends on the firing of GABA-releasing interneurons. In cortical microcircuits these interneurons display great diversity regarding their morphology, discharge properties, and synaptic wiring (Rudolph et al., 2001; Sieghart and Sperk, 2002; Fritschy and Brünig, 2003; Whiting, 2003; Möhler, 2006). Furthermore, different classes of GABAergic interneurons mediate their inhibitory action on excitatory PCs via different GABA_A_ receptor subtypes, structural properties that are well-preserved in OTC (Di Cristo et al., 2004). In the cortex, fast-spiking parvalbumin positive basket cells (FS) constitute a major class of GABAergic interneurons. They are excited by pyramidal neurons and in turn release GABA onto α1-containing GABA_A_ receptors located at high densities on the somata of PCs (Fritschy and Brünig, 2003). Thus, the activation of α1-receptors on PCs via FS-cells is part of a powerful inhibitory feedback loop. Unlike other GABAergic interneurons, action potential firing of FS-cells requires a strong excitatory drive, which is provided by PCs immediately after the onset of up states. Furthermore, FS-cells undergo prominent self-inhibition via autapses, a mechanism limiting the time for GABA being released by these neurons and activating α1-containing GABA_A_ receptors on pyramidal neurons (Bacci et al., 2005). The tight coupling between action potential firing of excitatory PCs, transient firing of inhibitory FS-cells, and subsequent activation of α1-containing GABA_A_ receptors may be the mechanism linking zolpidem’s α1-mediated effects to highly active network states (Figure 9).

### Actions of Zolpidem During Phases of Moderate Neuronal Activity

In the current study, peak firing rates of cortical neurons occurred within a narrow window of about 0 – 50 ms shortly after up state onset. Thereafter, action potential generation relaxed to a plateau of roughly 100 Hz. During this period of moderate neuronal activity, zolpidem significantly reduced its discharge rates. However, this effect was indistinguishable in wild type and α1(H101R) cultures. Our finding that zolpidem did not exclusively act via α1-containing GABA_A_ receptors in phases of moderate neuronal activity is consistent with a behavioral study exploring the drugs’ actions on the sleep time and EEG in wild type and α1(H101R) mice *in vivo* (Kopp et al., 2004). In this investigation zolpidem prolonged non-REM-sleep duration in wild type and α1(H101R) knock-in mice to a similar extent and the authors concluded that the hypnotic action of the drug could be mediated by α2- and/or α3-GABA_A_ receptors (Kopp et al., 2004). However, to clearly identify non-α1 GABA_A_ receptors targeted by zolpidem, additional studies on α2, α3, and α5 knock-in mice would be required.

### Different Benzodiazepine-Sensitive GABA_A_ Receptors Control Differential Forms of Neuronal Activity

We previously investigated the effects of the experimental α5-containing GABA_A_ receptor-preferring benzodiazepine SH-053-2′F-R-CH3 in cultured slices prepared from wild type and GABA_A_ receptor α5(H105R) knock-in mice (Drexler et al., 2013). Effects on firing patterns of cortical neurons elicited via α5-containing GABA_A_ receptors differed both from zolpidem’s α1-mediated actions and from the effects of SH-053-2′F-S-CH3. Enhancing the function of α5-containing GABA_A_ receptors did not alter peak discharge rates, but boosted action potential firing during phases of moderate neuronal activity. Unlike α1-containing GABA_A_ receptors, α5-containing GABA_A_ receptors are present in dendrites, but not in somata of PCs (Prenosil et al., 2006) and, as previously shown, in GABA-releasing interneurons (Rodgers et al., 2015), which may account for these differences. Overall, these findings suggest that those benzodiazepine-sensitive GABA_A_ receptors containing α1 are most effective in reducing high frequency action potential firing.

In summary, the findings from the current study may open novel ways to evaluate the use of drugs in clinical settings commonly associated with the occurrence of abnormal cortical hyperactivity, e.g., in patients suffering from traumatic brain injury or organophosphorus poisoning. In addition, we describe a system that may be used to evaluate pharmacological activity of substances at α1-containing GABA_A_ receptors, using native receptors. This may support the screening process for activity in α1-containing GABA_A_ receptors to also identify α1-sparing, i.e., non-sedative benzodiazepine-ligands which may be used to treat chronic pain, or in anxiety research and the treatment thereof. Furthermore, it is tempting to speculate that anesthetic agents which are highly effective in enhancing GABAergic inhibition via α1-containing GABA_A_ receptors may minimize the incidence of intraoperative awareness by preventing states of high frequency cortical activity, e.g., induced by surgical stimulation.

## Author Contributions

EN collected and analyzed the data and prepared the manuscript. UR helped to design the study and wrote the manuscript. DK, GL, and JC synthesized SH-053-2′F-S-CH3 and helped to design the study. HH performed paired recordings and helped to analyze the data. BA and BD designed the study, analyzed the data, and wrote the manuscript.

## Conflict of Interest Statement

The authors declare that the research was conducted in the absence of any commercial or financial relationships that could be construed as a potential conflict of interest.

## Abbreviations

ACSF: artificial cerebrospinal fluid
CI: confidence interval
FS: fast-spiking cell
GABA: γ-amino-butyric acid
iqr: interquartile range
NMDA: N-methyl-D-aspartate
OTC: organotypic culture
PC: pyramidal cell
PETH: peri-event time histogram
